# Delay in Surgery for Ruptured Aortic Aneurysm in Hemodynamically Stable Patients: A Multicenter Retrospective Analysis

**DOI:** 10.7759/cureus.89294

**Published:** 2025-08-03

**Authors:** Teiko Kawahigashi, Jo Taisuke, Taro Shimizu, Naoko Isogai, Hidemitsu Ogino, Toshio Takagi, Kazunao Watanabe, Hideo Yasunaga, Jun Kawachi

**Affiliations:** 1 Department of General Internal Medicine, Shonan Kamakura General Hospital, Kamakura, JPN; 2 Department of Emergency Medicine, Tokyo Nishi Tokushukai Hospital, Tokyo, JPN; 3 Department of Respiratory Medicine, The University of Tokyo, Tokyo, JPN; 4 Department of Diagnostic and Generalist Medicine, Dokkyo Medical University Hospital, Mibu, JPN; 5 Department of Surgery, Shonan Kamakura General Hospital, Kamakura, JPN; 6 Department of Surgery, Narita Tomisato Tokushukai Hospital, Chiba, JPN; 7 Department of Surgery, Tokyo Nishi Tokushukai Hospital, Tokyo, JPN; 8 Department of Clinical Epidemiology and Health Economics, The University of Tokyo, Tokyo, JPN

**Keywords:** abdominal aortic aneurysms, emergency medicine physician, general and vascular surgery, physical diagnosis, thoracic aortic rupture

## Abstract

Introduction: Thoracic aortic aneurysm or abdominal aortic aneurysm (TAA/AAA) is a fatal surgical emergency, and time to surgery can be a key factor in improving survival outcomes in patients. In this study, we examined the association between systolic blood pressure on arrival and door-to-surgery time in patients with ruptured TAA/AAA, hypothesizing that patients with ruptured thoracic or abdominal aortic aneurysms without hypotension may have longer door-to-surgery times than those with hypotension.

Methods: This retrospective study was conducted at two community hospitals, Shonan Kamakura General Hospital and Tokyo Nishi Tokushukai Hospital, in Japan, and included patients with ruptured thoracic or abdominal aortic aneurysms who were admitted to these hospitals from 2010 to 2021. Door-to-surgery time was compared between patients with stable blood pressure (>90 mmHg) and those with hypotension (≤90 mmHg) using multivariable linear regression.

Results: Among 94 patients (median age 82 years), 29.8% were in hypotension on arrival. Hemodynamically stable patients had a longer door-to-surgery time than those with hypotension (152 vs. 95.5 min, p = 0.003). Multivariable analysis showed significantly shorter door-to-surgery times for patients with hypotension (percent difference, -26.3%; 95% CI, -48.7% to -4.0%).

Conclusion: Hemodynamically stable patients with ruptured TAA/AAA can have longer door-to-surgery times, leading to avoidable delays in successful treatment. This study underscores the need for heightened clinical vigilance among physicians, as a delay in treatment may lead to worse outcomes in these patients.

## Introduction

Thoracic aortic aneurysm or abdominal aortic aneurysm (TAA/AAA) is a fatal surgical emergency [1-5]. Without emergency surgery, the mortality rate of ruptured TAAs/AAAs is nearly 100% [1,4,5]. Approximately 50% of patients die before reaching the hospital, while the overall mortality is estimated to be approximately 30% for open surgical repair and 17.9% for endovascular repair [1-6].

Time to surgery can be a key factor in improving survival outcomes. Studies have reported that timely diagnosis and immediate surgical repair could prevent death, whereas delays in diagnosis and treatment may worsen the prognosis [1-8]. A previous study reported a mortality rate of 73% in patients who underwent surgery more than two hours after onset, compared to 48% in those who underwent surgery within two hours [6,9]. Moreover, diagnostic delays can lead to preoperative shock, which is reported to be associated with increased mortality [10,11]. Another study reported higher mortality in patients with a ruptured AAA who were stable at presentation but developed hypotension before aortic cross-clamping than in those who remained stable or were already hypotensive at admission [12]. Thus, prompt diagnosis and surgical treatment are important for a better prognosis.

However, the diagnosis of a ruptured TAA/AAA can be challenging, and misdiagnosis at presentation is common (16%-60%) [13-18]. The classical triad (abdominal and/or back pain, a pulsatile abdominal mass, and hypotension) is considered diagnostic [2,3], but this triad may not manifest in its entirety. A previous study reported that more than half were hemodynamically stable, and misdiagnosis was significantly more common in these patients than in patients presenting with shock [11-18]. Misdiagnosis is significantly associated with delayed treatment [7], resulting in a poor prognosis.

Because hypotension is a key diagnostic indicator of a ruptured TAA/AAA, its absence may contribute to delays in diagnosis and treatment. However, only a few studies have thoroughly investigated this issue, and the existing ones are limited by small sample sizes and outdated data. To address these limitations and provide more robust evidence, we performed a retrospective study using a larger and more recent dataset to examine the relationship between blood pressure at hospital arrival and door-to-surgery time.

## Materials and methods

Study design and settings

This retrospective observational study was conducted at the emergency departments of two community hospitals in Japan (Shonan Kamakura General Hospital and Tokyo Nishi Tokushukai Hospital). Patient data were de-identified in each hospital, and the researchers were able to access anonymous data. This study was performed in accordance with the Declaration of Helsinki, and was approved by the Ethics Committee of Shonan Kamakura General Hospital, a core hospital in this study. Due to the retrospective nature of the study, the need for informed consent was waived by the Ethics Committee.

**Figure 1:**
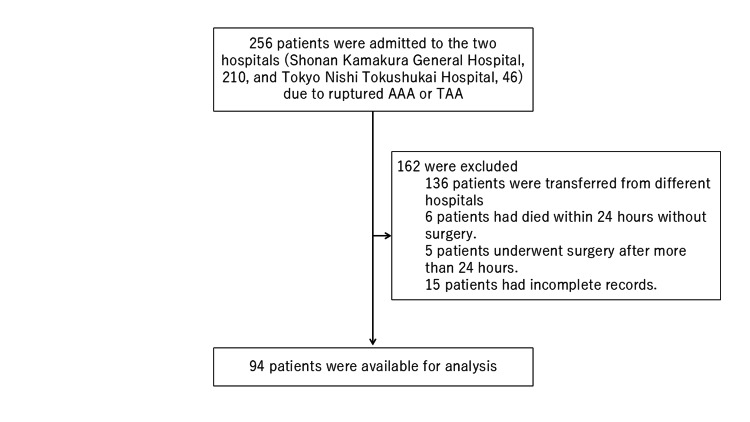
Flow diagram for patient recruitment AAA, abdominal aortic aneurysm; TAA, thoracic aortic aneurysm

We reviewed the medical records of patients with ruptured TAAs/AAAs who arrived at the hospitals between January 1, 2010, and December 31, 2021. Patients with incomplete records and those who were transferred from different hospitals were excluded. We accessed the data on March 23, 2023. Data were collected without any identifiable patient information. Figure 1 shows the flow diagram for the recruitment of participants for this study.

We collected data on patient demographics, including age, sex, preoperative symptoms (abdominal, chest, and back pain), comorbidities (hypertension, ischemic heart disease, and chronic kidney disease), smoking habit, and arrival during on-call time. We defined hypotension as systolic blood pressure ≤90 mmHg and stable blood pressure as systolic blood pressure >90 mmHg. Chronic kidney disease was defined as the presence of an abnormality in the kidney structure or function persisting for more than three months. Regular working hours were defined as 8:30 am to 5:00 pm on weekdays and 8:30 am to 1:00 pm on Saturdays. On-call time included all other hours. We also excluded patients who died within 24 hours of hospital arrival and those who underwent surgical repair after 24 hours since hospital arrival.

Outcome

The primary outcome was the door-to-surgery time, defined as the time from hospital arrival to the start of any surgical intervention, including both open repair and endovascular procedures.

Analysis

Continuous variables are presented as the means and standard deviations or median and interquartile range (IQR), and categorical variables are presented as numbers and percentages. Median door-to-surgery time was compared between the group with stable blood pressure (>90 mmHg) and the group with hypotension (≤90 mmHg) using the Mann-Whitney U test, and 28-day mortality was compared between the groups using the chi-square test.

We conducted a multivariable linear regression analysis to evaluate the association between door-to-surgery time and hypotension, based on blood pressure on arrival, with adjustment for possible confounders. The following confounding variables were chosen based on the literature: age [10,11,19,20], sex [20], abdominal pain and chest pain [2,3], comorbidities (hypertension [21], cardiovascular disease [11,22,23], and chronic kidney disease [10]), and arrival during on-call time [24,25]. Natural log transformation was applied to door-to-surgery time for homoscedasticity in linear regression. The coefficients of the regression analysis indicated the percentage difference (and 95% confidence interval) in door-to-surgery time for each independent variable. For the regression model, we excluded patients with high outliers of door-to-surgery time (>600 min).

All statistical analyses were conducted using the R software, version 4.0.3, for Windows (R Foundation for Statistical Computing, Vienna, Austria). All statistical tests were two-sided, and a p-value <0.05 was considered statistically significant.

## Results

During the study period, 256 patients with ruptured TAA/AAA were admitted, including 210 patients at Shonan Kamakura General Hospital and 46 patients at Tokyo Nishi Tokushukai Hospital. We excluded 15 patients with incomplete records, 136 patients transferred from different hospitals, 5 patients who underwent surgery after 24 hours, and 6 patients who died within 24 hours without surgery. Ultimately, 94 patients were included in this study.

The median age was 79 years (range, 42-98 years), and 63 patients were men (67.0%). Among the included patients, 69 (73.4%) had ruptured AAAs, 24 (25.5%) had ruptured TAAs, and 1 (1.1%) had a thoracoabdominal aortic aneurysm. On hospital arrival, 28 patients (29.8%) presented with hypotension. The baseline characteristics of the patients with a ruptured TAA/AAA with or without hypotension are presented in Table 1. Of the 94 patients, 69 (73.4%) complained of pain, with abdominal pain being the most frequent (38 patients, 40.4%). The most frequent comorbidity was hypertension (65.6%). Overall, 87 patients (92.6%) underwent surgical repair of the ruptured TAA/AAA. The median door-to-surgery time was 130 min (IQR, 72.5-220.5). The overall 28-day mortality was 34.0%.

**Table 1:** Patient characteristics Diagnosis: abdominal aortic aneurysm (AAA, N=69), thoracic aortic aneurysm (TAA, N=24), and thoracoabdominal aortic aneurysm (N=1)

Variable	All patients (N=94)	Stable blood pressure (>90 mmHg) (N=66)	Hypotension (≤90 mmHg) (N=28)
Age, years, median (interquartile range)	79 (42–98)	79 (42–98)	80 (62–98)
Male sex, n (%)	63 (67.0)	43 (65.2)	20 (71.4)
Symptoms, n (%)			
Abdominal pain	38 (40.4)	25 (37.9)	13 (46.4)
Back pain	35 (37.2)	30 (45.5)	5 (17.9)
Chest pain	12 (12.8)	12 (18.2)	0 (0.0)
Any pain	69 (73.4)	51 (77.3)	18 (64.3)
Medical history, n (%)			
Hypertension	61 (64.9)	43 (65.2)	18 (66.7)
Dyslipidemia	23 (24.5)	17 (25.8)	6 (21.4)
Diabetes mellitus	9 (9.6)	4 (6.1)	5 (17.9)
Cardiovascular disease	30 (31.9)	22 (33.3)	8 (28.6)
Chronic kidney disease	10 (10.6)	8 (12.1)	2 (7.1)
Chronic obstructive pulmonary disease	5 (5.3)	1 (1.5)	4 (14.3)
Smoking habit, n (%)	36 (48.3)	27 (48.2)	9 (50.0)
First visit to the emergency department, n (%)	88 (93.6)	60 (90.9)	28 (100.0)
Arrival during on-call time, n (%)	59 (62.8)	37 (56.1)	22 (78.6)

Unadjusted analysis of the outcomes is presented in Table 2. Patients who were hemodynamically stable on arrival had a significantly longer door-to-surgery time than those who were hypotensive (152 min vs. 95.5 min, p = 0.003). Patients with hypotension had a higher 28-day mortality rate than hemodynamically stable patients, but the difference was not statistically significant (46.4% vs. 28.8%, p = 0.10).

**Table 2:** Patient outcomes Median door-to-surgery times were compared using the Mann-Whitney U test, and 28-day mortality rates were compared using the chi-square test. Two-sided tests were used. p-values <0.05 were considered statistically significant.

Outcome	All patients (N=94)	Stable BP (>90 mmHg) (N=66)	Hypotension (≤90 mmHg) (N=28)	p-value
Door-to-surgery time, median (interquartile range)	130 (72.5–220.5)	152 (99–305)	96 (55–146)	0.003
28-day mortality, n (%)	32 (34.0)	19 (28.8)	13 (46.4)	0.1

**Figure 2:**
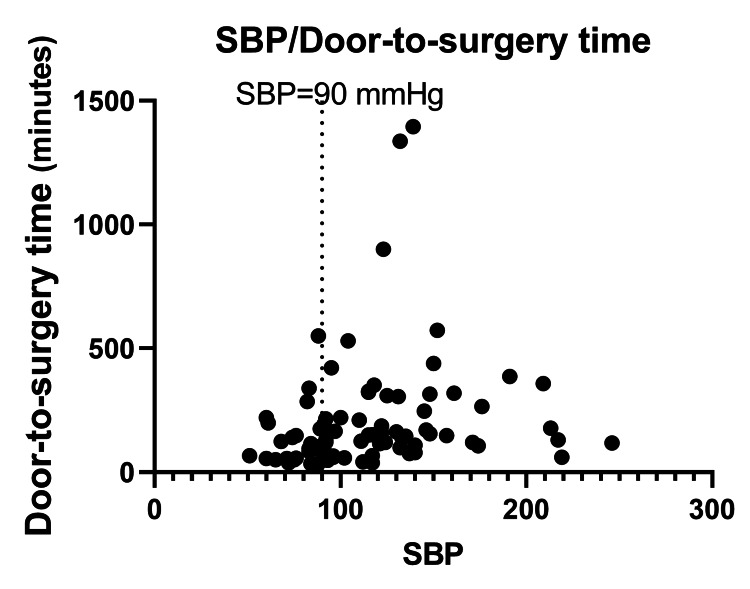
Scatter plot for the systolic blood pressure (SBP) on arrival and door-to-surgery time

Figure 2 shows a scatter plot for systolic blood pressure on arrival and door-to-surgery time. We excluded three patients from the regression analysis with a door-to-surgery time of >600 min. The multivariable linear regression analysis revealed that door-to-surgery time was 26.3% (95% confidence interval, 48.7% to 4.0%) shorter in patients with hypotension compared to stable patients (Table 3). No other factors were associated with differences in door-to-surgery time.

**Table 3:** Multivariable linear regression analysis for door-to-surgery time The analysis was performed using multivariable linear regression. Percent differences and 95% confidence intervals are shown. p-values <0.05 were considered statistically significant.

	Percent difference	95% Confidence interval		p-value
Hypotension (versus stable blood pressure)	-26.3%	-48.7	-4.0	0.022
Age (years)	12.2%	-10.4	34.3	0.289
Male (versus female)	-11.3%	-36.5	13.2	0.354
Abdominal pain	-2.6%	-24.3	19.2	0.816
Chest pain	8.1%	-16.1	32.7	0.499
Hypertension	7.3%	-15.0	29.4	0.520
Cardiovascular disease	6.5%	-15.8	28.6	0.567
Chronic kidney disease	-7.3%	-29.8	15.8	0.544
Arrival during on-call time	-18.2%	-39.4	3.4	0.098

## Discussion

The present study revealed that patients with ruptured AAAs/TAAs without hypotension were more likely to have longer door-to-surgery times. Hypotension involves acute circulatory failure, alerting emergency physicians and staff to the need for immediate intervention. In contrast, patients without hypotension may not be promptly recognized as critical, potentially leading to delayed treatment.

Considering the findings of the present study along with the previous research, it is suggested that classic signs such as abdominal and/or back pain and hypotension may not always be present in patients with ruptured aortic aneurysms. In our cohort, only 69 of the 94 patients (73.4%) reported pain, and just 30% were hypotensive upon arrival. These findings align with previous studies. A single-center study from the United States reported that 64% (16/25) of patients with ruptured infrarenal AAA had an initial systolic blood pressure above 90 mmHg [12]. Similarly, a study from the United Kingdom found that 57.1% of emergency AAA patients were normotensive on admission [2].

The unadjusted analysis revealed that patients without hypotension had a 56-min longer door-to-surgery time. Multivariable regression revealed that patients with hypotension had approximately 26% shorter door-to-surgery time than those without hypotension. This delay in patients without hypotension can result in a poor prognosis. Many previous studies have shown that preoperative hypotension is one of the most important predictors of in-hospital mortality in patients with AAA [26-29]. Our findings are consistent with these reports. In our study, patients who were hypotensive on admission tended to have higher mortality, although the difference was not statistically significant.

Several factors may contribute to delayed surgery, among which a low level of awareness among medical staff may play a role. In other words, while clinicians may be highly alert to the possibility of AAA or TAA in unstable patients, their level of attention may decrease when patients present with stable vital signs. This may result in a delay in reaching the correct diagnosis (diagnostic delay) or, in some cases, an incorrect diagnosis (misdiagnosis). In this study, we did not assess the specific causes of delay, such as door-to-CT or diagnosis time. Therefore, it remains unclear whether diagnostic delay or misdiagnosis was the main contributor to the prolonged time to surgery. However, previous studies have reported that misdiagnosis occurred more frequently in patients with normal blood pressure than in those who were hypotensive [2,12]. A decrease in clinical vigilance may increase the likelihood of misdiagnosis, which in turn could lead to further diagnostic delays.

There may be several sources of bias. For example, in our cohort, patients with hypotension tended to present more frequently during on-call hours, although this trend was not statistically significant. Interestingly, patients who arrived during on-call hours also had shorter door-to-surgery times, again without reaching statistical significance. One possible explanation is that, during regular hours, surgeons often need to reschedule elective surgeries to accommodate emergency cases, whereas no such scheduling conflict exists during off-hours. This may contribute to a relatively shorter waiting time for emergency surgery during on-call periods.

Because patients without hypotension may have a greater potential for survival, early diagnosis and timely treatment are especially critical. A previous study reported that patients with emergency AAA who became hypotensive before aortic cross-clamping had higher mortality rates than those who were hemodynamically stable or already hypotensive at admission [12]. This may be because normotensive patients at presentation have a better physiological reserve, but a delayed treatment could lead to deterioration into hypotension, thereby worsening outcomes. Therefore, delays in treatment should be avoided to preserve lifesaving opportunities.

In addition to longer in-hospital delays, hemodynamically stable patients may also experience a prolonged interval from symptom onset to hospital arrival, potentially resulting in an overall delay in receiving surgical intervention. Although our study did not analyze the time from symptom onset to hospital arrival, previous research has shown that patients with stable vital signs tend to have significantly longer symptom duration compared to hypotensive patients [2]. These findings underscore the need for heightened clinical vigilance, as delayed treatment in this patient population may contribute to worse outcomes.

Cognitive biases may be one of several contributing factors to delays in diagnosis and treatment. In emergency care settings, where high workload and time pressure are common, biases such as anchoring, premature closure, availability, and hassle bias can influence decision-making [30]. While emergency physicians must promptly identify critically ill patients, it can be especially challenging to reassess those with initially mild symptoms. Educating clinicians that hemodynamic stability does not rule out serious illnesses and promoting reflective practice may help reduce diagnostic delays.

The present study had several limitations. First, the unblinded data were collected retrospectively; therefore, observer bias cannot be excluded. Second, we only included patients who underwent surgery for AAAs/TAAs. Due to a lack of data, the study population did not include those who died without a diagnosis or who were diagnosed at autopsy. Third, although we analyzed TAAs and AAAs together, these two conditions differ in terms of pathophysiology, clinical presentation, and prognosis. Therefore, the findings should be interpreted with consideration of these differences. Future studies focusing on each condition separately may help to further clarify the observed trends. Fourth, this study did not collect information on the development of hypotension after hospital arrival, which may have influenced clinical decision-making and outcomes. Further investigations including such data are warranted to better understand these effects.

## Conclusions

In conclusion, hemodynamically stable patients with a ruptured AAA/TAA had longer door-to-surgery times than those with hypotension. This may be due to reduced clinical vigilance in seemingly stable patients. Physicians need to have a higher level of awareness of a ruptured AAA/TAA even in patients without hypotension because they potentially have a higher chance of survival than patients with hypotension if prompt diagnosis and appropriate treatment are provided.
